# Supplementary material for: Complete Depletion of Daratumumab Interference in Serum Samples from Plasma Cell Myeloma Patients Improves the Detection of Endogenous M-Proteins in a Preliminary Study
Source: Diagnostics (Basel). 2020 Apr 14;10(4):219. doi: 10.3390/diagnostics10040219 (PMC7235820; doi:10.3390/diagnostics10040219)
Supplement: Supplementary file 1 [file diagnostics-10-00219-s001.pdf]

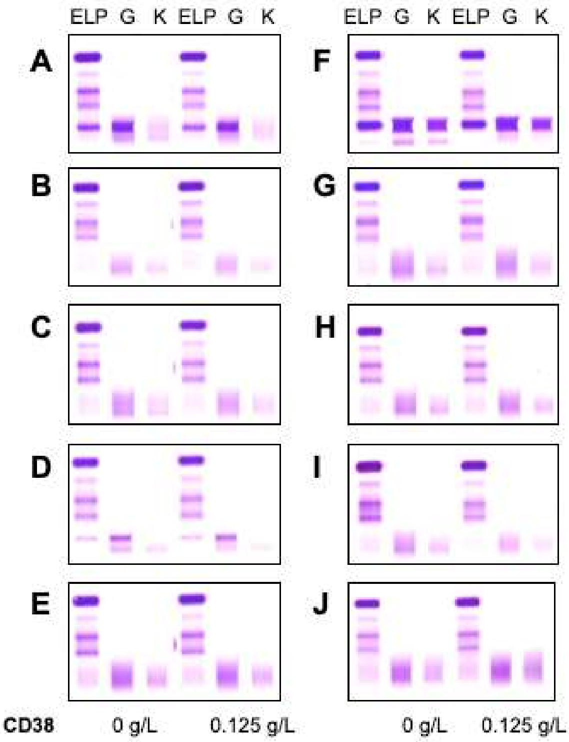

Sample	Original M-protein
A	IgG lambda
B	IgD lambda and free lambda light chains
C	IgA kappa
D	IgG lambda and IgG kappa
E	IgA kappa
F	IgG kappa
G	IgG kappa and free lambda light chains
H	IgA kappa
I	IgA lambda
J	IgG kappa

Figure S1. The impact of pre-treatment and efficiency of recombinant biotinylated CD38 tested in sera from patients with PCM who were receiving DARA as a therapy.

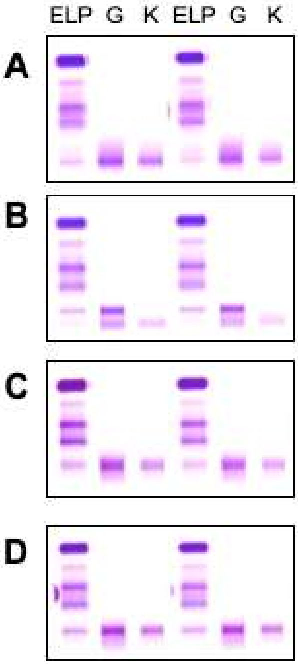

Sample	Original M-protein
A	IgG kappa
B	IgG lambda and IgG kappa
C	IgG kappa
D	IgG kappa

Figure S2. Representatives of PCM patients with endogenous IgG kappa receiving daratumumab therapy where daratumumab comigrates with the endogenous M-protein and CD-38 treatment had no effect on the endogenous IgG kappa.
